# Facing PHACES Syndrome; Anesthesiologist’s Point of View

**DOI:** 10.5812/aapm-141896

**Published:** 2024-01-11

**Authors:** Dariush Abtahi, Alireza Shakeri, Ardeshir Tajbakhsh

**Affiliations:** 1 Anesthesia Research Center, Shahid Beheshti University of Medical Sciences, Tehran, Iran; 2 Imam Hossein General Hospital, Shahid Beheshti University of Medical Sciences, Tehran, Iran

**Keywords:** Anesthesia, Pediatrics, PHACES Syndrome

## Abstract

**Introduction:**

PHACES syndrome is a neurocutaneous syndrome that consists of posterior fossa brain malformation, hemangiomas of the face, arterial cerebrovascular malformation, cardiovascular anomalies (Coarctation of the aorta), eye anomaly, and sternal defect or supraumbilical raphe. Most of these manifestations would interfere with anesthesia and hence need major considerations.

**Case Presentation:**

A 2.5-month-old female weighing 2100 gr was a candidate for laser therapy due to retinopathy of prematurity. She was diagnosed with PHACES syndrome. Her anesthesia was induced and maintained with sevoflurane. LMA #1 was used for airway management. No complications occurred during or after anesthesia, and she was discharged the next day.

**Conclusions:**

PHACES syndrome interacts with numerous anesthesia-related characteristics, such as airway management, cardiovascular disease, and CNS malformations. Based on the accompanying characteristics, perioperative care for these patients should be tailored.

## 1. Introduction

Infantile Hemangioma is the most common benign tumor in pediatrics. Its incidence ranges from 5% to 10% (1). It has been reported that 2% to 3% of these patients suffer from PHACES syndrome (1). PHACES syndrome (OMIM no. 606519) was described in 1996 by Frieden et al. (2). It is a neurocutaneous syndrome and an acronym for Posterior fossa brain malformation, Hemangiomas of the face, Arterial cerebrovascular malformation, Cardiovascular anomalies (Coarctation of the aorta), Eye anomaly, Sternal defect or supraumbilical raphe (3). Although cases with all six components are reported to be rare, about 20% of cervicofacial hemangiomas in infancy are associated with some features of PHACES syndrome (1). The most common extracutaneous manifestations are cerebrovascular anomalies (87%) followed by cardiovascular (37%), eye (16%), ventral developmental defects (7 - 21%), and endocrine abnormalities (6%). Other accompanying malformations are micrognathia, auricular hypoplasia, supra and subglottic hemangiomas, and pituitary insufficiency. As the female-to-male ratio is reported to be 8: 1 to 10: 1, this syndrome is considered an X-linked dominant disorder that causes spontaneous abortion of a male fetus (4, 5).

Its prognosis is reported to be variable and mostly depends on the extent of cardiovascular anomalies and arterial cerebrovascular malformations. Although the features of this syndrome are defined, a longitudinal study of these populations has not yet been defined (6). Management of this syndrome ranges from observation to numerous complex surgical procedures. As many of these patients require surgery and anesthesia, it should be noted that anesthesia in these patients poses different risks and considerations. 

Although more than 250 case reports and series have been published, there is scarce evidence for their anesthesia management. Neurologic pathologies along with cardiac anomalies predispose these patients to stroke and seizure. On the other hand, supra and subglottic hemangiomas pose anesthesiologists with difficulty in intubation, airway obstruction risk, and airway bleeding (7).

## 2. Case Presentation

**Figure 1.**
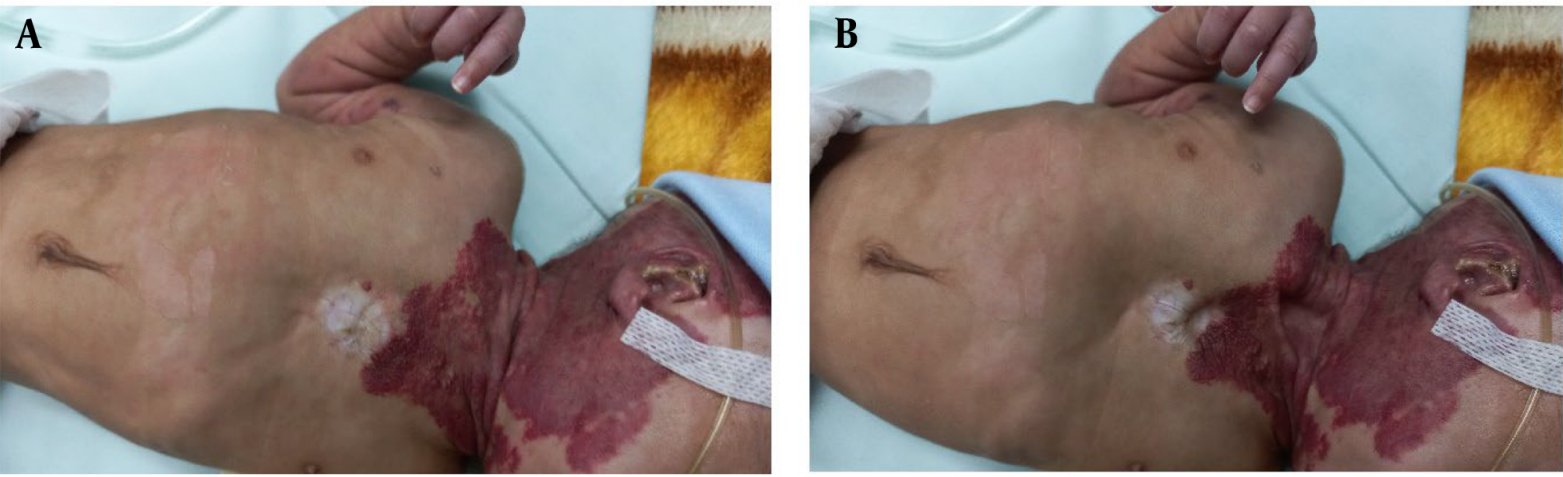
A, during exhalation; B, during inspiration

A 2.5-month-old female delivered by cesarean section at 28 weeks gestational age due to placenta previa weighing 1500 g was eligible for laser treatment with a retinopathy of prematurity (ROP) diagnosis. Her weight at surgery was 2100 grams, and she was diagnosed with PHACES syndrome. Her diagnosis was based on extensive hemangioma on her neck and chest with a cleft in her lower lip and supraumbilical raphe (Figure 1). A preoperative bronchoscopy was performed and revealed a subglottic and tracheal hemangioma, causing 25% to 50% stenosis without dyspnea along with choanal stenosis. Her echocardiogram revealed a 4 mm perimembranous VSD. An MRI examination of the brain was normal. She was treated with propranolol, captopril, dexamethasone, and sirolimus. Her nasal cannula was also used to ensure oxygen delivery. Laboratory tests showed her hemoglobin level to be 8.6 g/dl and her TSH level to be within the normal range. An epinephrine nebulizer was prescribed the day before surgery to minimize the effects of the hemangioma on the airways.

Anesthetic induction was facilitated through the administration of sevoflurane with spontaneous respiration, gradually increasing from 1% to 6% in the presence of 100% oxygen. Meanwhile, the patient was monitored with ECG, Pulse Oximetry, and Respiratory rate. Subsequently, a size #1 laryngeal mask airway (LMA) was inserted successfully following confirmation of profound anesthesia through clinical evaluations encompassing respiratory patterns, pupillary alignment, and heart rate. In light of the expected compromised airway, the use of neuromuscular blocking agents was intentionally avoided. For the maintenance phase during laser therapy, sevoflurane at a concentration of 3% was administered using a Mapleson type D system. Upon completion of the procedure, sevoflurane administration was ceased. The LMA was removed while the patient remained in a deep anesthetic state, and an oxygen mask was employed until full emergence from anesthesia. Subsequently, the patient was discharged the day following surgery without encountering any postoperative complications.

## 3. Discussion

After the introduction of PHACES syndrome in 1996, diagnostic criteria were developed in 2009 that categorized patients with possible or definite diagnoses (Table 1) (8). The most common feature would be large and segmental hemangiomas, which are more than 5 cm in diameter (8). Our case has hemangioma > 5 cm in the scalp, neck, and upper trunk, without any CNS or arterial abnormality. It also has a small VSD, ROP, supraumbilical raphe, and sternal defect. Based on revised criteria, our case was a definite PHACES syndrome with Hemangioma > 5 cm plus one major and one minor criterion. We do not attribute ROP to this syndrome as it may develop due to oxygen therapy after preterm labor. Our case had another ventral defect, a lower lip cleft, which was not reported in previous studies.

**Table 1.** Diagnostic Criteria Revised in 2009 (8)

Acronym	Organ System	Major Criteria	Minor Criteria
**Posterior fossa brain malformation**	Structural brain	Posterior fossa brain anomalies, dandy-walker complex, other hypoplasia/dysplasia of the mid and/or hindbrain.	Midline brain anomalies, malformation of cortical development.
**Arterial cerebrovascular malformation**	Arterial anomalies	Anomaly of major cerebral or cervical arteries ^a^, dysplasia ^b^ of the large cerebral arteries, arterial stenosis or occlusion with or without moyamoya collaterals, absence or moderate-severe hypoplasia of the large cerebral and cervical, arteries, aberrant origin or course of the large cerebral or cervical arteries except, common arch variants such as bovine arch., persistent carotid-vertebrobasilar anastomosis (proatlantal segmental,hypoglossal, otic, and/or trigeminal arteries).	Aneurysm of any of the cerebral arteries.
**Cardiac abnormality/ coarctation of the aorta**	Cardiovascular	Aortic arch anomalies, coarctation of the aorta, dysplasia ^a^, aneurysm, aberrant origin of the subclavian artery with or without a vascular ring.	Ventricular septal defect, right aortic arch/double aortic arch, systemic venous anomalies.
**Eye anomalies**	Ocular	Posterior segment abnormalities, persistent hyperplastic primary vitreous, persistent fetal vasculature, retinal vascular anomalies, morning glory disc anomaly, optic nerve hypoplasia, peripapillary staphyloma.	Anterior segment abnormalities, microphthalmia, sclerocornea, coloboma, cataracts.
**Sternal defect/supraumblical raphe**	Ventral/midline	Anomaly of the midline chest and abdomen, -sternal defect, -sternal pit, -sternal cleft, -supraumbilical raphe.	Ectopic thyroid hypopituitarism, midline sternal papule/hamartoma.
**Definite PHACE**			
Hemangioma > 5 cm in diameter of the head, including scalp, plus 1 major criteria or 2 minor criteria		Hemangioma of the neck, upper trunk or trunk, and proximal upper extremity, plus 2 major criteria.	
**Possible PHACE**			
Hemangioma > 5 cm in diameter of the head, including scalp, plus 1 minor criteria		Hemangioma of the neck, upper trunk or trunk, and proximal upper extremity, plus 1 major or 2 minor.	No hemangioma, plus 2 major criteria.

^a^ Internal carotid artery, middle cerebral artery, anterior cerebral artery, posterior cerebral artery, or vertebrobasilar system.

^b^ Includes kinking, looping, tortuosity, and/or dolichoectasia.

When PHACES syndrome is considered, initial screening should include a physical examination, echocardiography, MRI/MRA of the head and neck, and an ophthalmology exam.

Posterior fossa malformations, like the Dandy-Walker complex, may endanger these patients to brain herniation upon a rise in Intracranial Pressure (ICP) (9). Therefore, anesthesiologists should be aware of this anomaly to prevent any increase in ICP.

Hemangiomas may endanger the airway. These patients are at an increased risk of airway obstruction due to hemangiomas (2). We recommend performing a flexible bronchoscopy or HRCT of the trachea before any attempt to intubate these patients.

Propranolol has been extensively employed in the treatment of patients afflicted with infantile hemangiomas (10). The administration of propranolol may result in bradycardia within the cardiovascular system, necessitating the consideration of atropine therapy in the event of bradycardia occurrence, particularly in infants whose cardiac output is reliant upon heart rate. Conversely, the routine administration of atropine for pediatric airway management is not advised (11). In our particular case, the patient exhibited a heart rate ranging from 120 to 130 beats per minute, rendering the prescription of atropine unnecessary.

Guidelines about perioperative adrenal insufficiency in pediatric patients advocate for the uninterrupted continuation of oral medication during surgical procedures (12). Even though our neonate was not considered to have adrenal insufficiency due to the brevity and minimal dosage of corticosteroid administration, there remains a recommendation to contemplate the administration of corticosteroids for those individuals necessitating major surgery or with a history of prolonged corticosteroid utilization.

Arterial cerebrovascular malformations may threaten these patients with cerebral ischemia during anesthesia (2). Therefore, careful hemodynamic management to maintain cerebral perfusion pressure is mandatory.

Cardiac abnormalities might be exaggerated or complicated by anesthetic agents (13). Therefore, echocardiography should be done before any surgery, and anesthetic techniques should be tailored for these patients. 

In the operating room, care should be taken to minimize sympathetic activity. This may cause enlargement of hemangiomas and increase the risk of airway occlusion. Also, if a long procedure is estimated, brain oxygenation monitoring, e.g., rSO2 or NIRS, would be rationale (2, 13).

Postoperative care should include close hemodynamic monitoring, and seizures might occur during this period (5). Table 2 summarizes anesthetic implications in these patients.

**Table 2.** Anesthetic Manifestations of PHACES Syndrome

Manifestations	Anesthetic Implications
**Posterior fossa malformations**	Avoid any increase in ICP
**Hemangiomas**	Difficult Intubation
**Arterial cerebrovascular malformations**	Maintain CPP to avoid brain ischemia
**Cardiovascular anomaly**	Tailored anesthetic drugs for any anomalies
**Eye anomaly**	Preoperative eye examination
**Sternal cleft**	Assessment for restrictive pulmonary diseases

### 3.1. Conclusions

As PHACES syndrome can interfere with anesthesia through different manifestations, special consideration is mandatory when facing this syndrome. Therefore, a comprehensive preoperative evaluation for assessment of CNS, cardiac anomalies, and airway should be noted.

## Data Availability

The dataset presented in the study is available on request from the corresponding author during submission or after publication.
